# Analysis of Clinical Features, Diagnostic Tests, and Biomarkers in Patients With Suspected Creutzfeldt-Jakob Disease, 2014-2021

**DOI:** 10.1001/jamanetworkopen.2022.25098

**Published:** 2022-08-03

**Authors:** Dror Shir, Evelyn B. Lazar, Jonathan Graff-Radford, Allen J. Aksamit, Jeremy K. Cutsforth-Gregory, David T. Jones, Hugo Botha, Vijay K. Ramanan, Christian Prusinski, Amanda Porter, Gregory S. Day

**Affiliations:** 1 Department of Neurology, Mayo Clinic, Rochester, Minnesota; 2 Department of Neurology, Mayo Clinic, Jacksonville, Florida

## Abstract

**Importance:**

Detection of prion proteins in cerebrospinal fluid (CSF) using real-time quaking-induced conversion (RT-QuIC) assays has transformed the diagnostic approach to sporadic Creutzfeldt-Jakob disease (CJD), facilitating earlier and more complete recognition of affected patients. It is unclear how expanded recognition of affected patients may affect the diagnostic and prognostic relevance of clinical features and diagnostic tests historically associated with CJD.

**Objective:**

To evaluate clinical features and diagnostic testing in patients presenting with CJD and determine the associations of these features with prognosis.

**Design, Setting, and Participants:**

This cohort study incorporated data from electronic medical records of patients with CJD treated at Mayo Clinic Enterprise tertiary care centers in Rochester, Minnesota; Jacksonville, Florida; and Scottsdale, Arizona. Participants included patients with definite or probable CJD assessed from 2014 to 2021. Data were analyzed October 2021 to January 2022.

**Exposures:**

Dominant presentation, clinical features, and diagnostic tests associated with CJD.

**Main Outcomes and Measures:**

The outcomes of interest were the sensitivity and prognostic value of clinical features and accessible diagnostic tests at presentation with possible CJD.

**Results:**

A total of 115 patients were identified, including 40 patients (35%) with definite CJD. Mean (SD) age at symptom onset was 64.8 (9.4) years, and 68 patients were women (59%). The sensitivity of clinical markers (myoclonus) and tests historically considered in patients with suspected CJD was poor (eg, stereotyped electroencephalography anomalies: 17 of 105 patients [16%]; elevated CSF protein 14-3-3 levels: 54 of 90 patients [60%]). By comparison, biomarkers with good diagnostic sensitivity at presentation included RT-QuIC (66 of 71 patients [93%]), CSF total tau (T-tau) level greater than 1149 pg/mL (81 of 92 patients [88%]), and characteristic signal anomalies on magnetic resonance imaging (88 of 115 patients [77%]). Multivariable linear regression confirmed shorter survival in patients with myoclonus (difference, −125.9 [95% CI, −236.3 to −15.5] days; *P* = .03), visual or cerebellar signs (difference, −180.2 [95% CI, −282.2 to −78.2] days; *P* < .001), elevated CSF protein 14-3-3 levels (difference, −193 [95% CI, −304.9 to −82.9] days; *P* < .001), and elevated T-tau level (difference for every 1000 pg/mL elevation, −9.1 [95% CI, −17.7 to −1.0] days; *P* = .04).

**Conclusions and Relevance:**

These findings suggest that CSF RT-QuIC, elevated CSF T-tau level, and stereotyped magnetic resonance imaging anomalies were associated with the diagnosis of CJD, while other clinical findings (eg, myoclonus), stereotyped electroencephalography anomalies, and CSF protein 14-3-3 levels offered less diagnostic value. Visual or cerebellar features, myoclonus, and CSF 14-3-3 and T-tau levels may be associated with disease duration, justifying continued inclusion in the evaluation of patients suspected to have CJD.

## Key Points

**Question:**

What is the diagnostic utility associated with clinical features and tests historically associated with Creutzfeldt-Jakob disease (CJD) in patients evaluated in the modern era?

**Findings:**

In this cohort study including 115 patients, the diagnostic performance of cerebrospinal fluid real-time quaking-induced conversion assays, total tau, and stereotyped signal anomalies on magnetic resonance imaging was high, while the diagnostic yield of myoclonus and periodic discharges on electroencephalography was low. The presence of myoclonus, visual or cerebellar findings, and elevated levels of protein 14-3-3 and total tau in cerebrospinal fluid were independently associated with shorter time to death.

**Meaning:**

These findings may guide prioritization and interpretation of accessible tests in patients with suspected CJD.

## Teaser

This cohort study evaluates clinical features and diagnostic testing in patients presenting with potential Creutzfeldt-Jakob disease and the associations of these features with prognosis.

## Introduction

Diagnostic criteria for sporadic Creutzfeldt-Jakob disease (CJD) have historically emphasized archetypal clinical features and established diagnostic biomarkers. Accordingly, the US Centers for Disease Control and Prevention (CDC) diagnostic criteria emphasize detection of a neuropsychiatric disorder or progressive dementia, as well as core clinical features (ie, visual or cerebellar disturbance, pyramidal or extrapyramidal dysfunction, and akinetic mutism) and myoclonus—a hallmark clinical sign.^1,2,3^ Diagnostic biomarkers include specific findings on brain magnetic resonance imaging (MRI; ie, hyperintense signal and reduced diffusivity involving cerebral cortex and corpus striatum^4,5,6,7^), characteristic electroencephalography (EEG) patterns (ie, periodic synchronous biphasic or triphasic sharp wave complexes^8,9^), and elevated cerebrospinal fluid (CSF) protein 14-3-3 level.^10,11,12^ The more recent discovery and validation of real-time quaking-induced conversion (RT-QuIC) assays capable of detecting malformed prion proteins in CSF has transformed the diagnostic approach to CJD, allowing the diagnosis of probable CJD to be established with higher confidence.^13^ Wide application of RT-QuIC testing and integration of results within diagnostic criteria have substantially improved recognition of affected patients, increasing prevalence estimates, broadening appreciation of the clinical phenotypes attributable to CJD, and permitting diagnoses to be established earlier in the clinical course.^14,15^

The diagnostic and prognostic relevance of previously established clinical features and biomarkers associated with CJD remains unclear and warrants reevaluation in the modern era. Recognizing this, we evaluated the diagnostic performance of key clinical features and widely available tests for CJD in patients with clinically probable or definite (pathology-proven) CJD diagnosed in the RT-QuIC era. Specifically, we sought to determine the frequency of clinical features and biomarker changes at initial evaluation and to understand the associations of clinical features and diagnostic tests with disease duration.

## Methods

This cohort study was approved by the Mayo Clinic institutional review board. A waiver of consent was granted from the Mayo Clinic institutional review board for the use of retrospective, deidentified data. This study followed the Strengthening the Reporting of Observational Studies in Epidemiology (STROBE) reporting guideline for cohort studies.

Mayo Clinic Enterprise database search software was used to identify patients diagnosed with CJD from January 2014 to September 2021 at Mayo Clinic in Rochester, Minnesota; Jacksonville, Florida; and Scottsdale, Arizona), as reported elsewhere.^16^ The study start date was selected to capture the time at which specific biomarkers of prion disease (specifically, RT-QuIC) were incorporated within routine clinical practice.

### Clinical Data

The initial search identified 269 patients with suspected CJD using diagnostic codes for CJD (eg, dementia due to prion disease, Creutzfeldt-Jakob disease, atypical virus infections of the central nervous system, of a possible 2 014 280 unique patients). Electronic medical records were screened by 2 of us (D.S. and E.B.L.) from April to October 2021 to identify patients who met criteria for probable CJD (neuropsychiatric disorder, including but not limited to rapidly progressive dementia with positive RT-QuIC; or rapidly progressive dementia with myoclonus, visual or cerebellar signs, pyramidal or extrapyramidal signs, akinetic mutism; and consistent brain MRI), or definite CJD (pathologically or genetically confirmed). Patients with genetic familial prion disease or sporadic fatal familial insomnia were excluded.

Relevant data were extracted from the electronic medical records, including demographics (age, sex), medical comorbidities, age at symptom onset, clinical features supportive of CJD diagnosis (citied within CDC Diagnostic Criteria^2^), and results of testing (MRI, EEG, CSF measures of 14-3-3 protein, total tau [T-tau] level, and RT-QuIC). Brain MRIs were initially read by radiologists as a part of the patients’ examinations, blinded to other test results, and prior to results of RT-QuIC test in all but 1 patient. MRIs were classified as consistent with CJD if high T2-signal was reported in caudate or putamen or at least 2 cortical regions (eg, temporal, parietal, occipital) on diffusion-weighted imaging or fluid attenuated inversion recovery. Images were directly reviewed by 2 of us (D.S. and G.S.D.) before concluding that MRIs were not consistent with CJD. CSF measures were performed by the National Prion Disease Pathology Surveillance Center at Case Western Reserve University. Based on data from previous literature and consistent with CJD according to The National Prion Disease Pathology Surveillance Center, the CSF T-tau cutpoint was greater than 1149 pg/mL.^15,17,18,19,20^ When available, death date and results of neuropathological evaluation and molecular subtyping were recorded.

Dominant initial presentations were retrospectively assigned by a neurologist (D.S.) based on reported symptoms and signs at presentation. Cognitive impairment presentations included patients with predominant language, memory, executive, visual, or auditory agnosia impairment. Motor impairment included patients with prominent pyramidal signs, extra-pyramidal signs, corticobasal syndrome–like presentation (eg, alien limb, dystonia, apraxia), or hyperkinetic movement disorder. Psychiatric impairment included patients with predominant behavioral change and/or psychosis. Global impairment included patients with near-simultaneous onset of symptoms across several neurologic domains (eg, cognitive, cerebellar, and motor). Cerebellar impairment included patients with predominant ataxic gait and/or dysmetria.

### Statistical Analysis

Demographic and clinical characteristics were summarized using descriptive statistics. Differences in the frequency of diagnostic features across dominant initial presentations were evaluated using Fisher exact test. The associations between survival (time from detection or test to death), and key clinical features and diagnostic biomarker results were examined using Kaplan-Meier survival analyses, with significance testing performed using the log-rank test. Multivariable regression analyses were used to further consider the associations of clinical features and CSF biomarkers with time-to-death, controlling for age and other significant associations. For survival analyses, first detection or test was used to determine association (1 observation per participant). For regression analyses, time-to-death was calculated from each observation or test (1 observation per test). Pearson ρ was used to assess the association between time from symptom onset and CSF T-tau level.

Statistical analyses were performed with SPSS statistical software version 28.0 (IBM). *P* values were 2-sided, with significance established at *P* < .05. Correction for multiple comparisons was not performed owing to the focus on testing of well-established features. Data were analyzed from October 2021 to January 2022.

## Results

### Participant Demographics

A total of 115 patients were identified, including 75 patients (65%) with probable CJD, and 40 (35%) with definite CJD. Mean (SD) age at symptom onset was 64.8 (9.4) years, and 68 patients (59%) were women. Table 1 summarizes participant demographics, clinical features, and outcomes. Patients presented a median (IQR) of 8.7 (2.3-21.9) weeks after symptom onset. The main concerns leading to neurological consultation were cognitive difficulties (66 patients [57%]), gait changes (53 patients [46%]), behavioral changes (26 patients [23%]), motor symptoms (14 patients [12%]), sensory concerns (22 patients [19%]), and visual concerns (19 patients [17%]). Cognitive presentations predominated (49 patients [43%]), while pure psychiatric presentations were rare (4 patients [3%]).

**Table 1.** Patient Demographics and Clinical Features

Characteristics	Patients, No. (%) (N = 115)
Sex	
Men	47 (41)
Women	68 (59)
Age of onset, mean (SD), y	64.8 (9.4)
Time from symptom onset to first assessment, median (IQR), wk	8.7 (2.3-21.9)
Dominant initial presentation	
Cognitive^a^	49 (43)
Global	28 (24)
Cerebellar	22 (19)
Motor	12 (10)
Psychiatric	4 (3)
Clinical features supportive of CJD diagnosis^b^	
Rapidly progressive dementia	111 (97)
Myoclonus	63 (55)
Visual or cerebellar signs	101 (88)
Pyramidal or extrapyramidal signs	50 (43)
Akinetic mutism	8 (7)
Outcome data	
Symptom duration, median (IQR), wk	34.9 (13.6-73.4)
Definite CJD^c^	40 (35)
Molecular subtype	
Any	35 (30)
MM1	12 (34)
MM1-2	2 (6)
MM2	2 (6)
MV1	4 (11)
MV1-2	5 (14)
MV2	2 (6)
VV1	1 (3)
VV1-2	1 (3)
VV2	6 (17)

Abbreviations: CJD, Creutzfeldt-Jakob disease; M, methionine; V, valine.

^a^ Cognitive includes amnestic, dysexecutive, language, visual, and auditory subtypes.

^b^ Features supportive of CJD diagnosis according to Diagnostic Criteria by the Centers for Disease Control and Prevention.^2^

^c^ Definite CJD includes pathologically or genetically confirmed CJD.

### Core Clinical Features and Diagnostic Biomarkers at Presentation

Table 2 details the sensitivity of clinical features and diagnostic biomarkers at initial presentation. No differences in sensitivity of clinical features or diagnostic test results were observed between patients with definite vs probable diagnoses. The most common clinical features at initial presentation were rapidly progressive dementia (90 of 115 patients [77%]), followed by visual or cerebellar signs (74 of 115 patients [64%]). Most patients exhibited CSF T-tau levels greater than 1149 pg/mL (81 of 92 patients [88%]; mean [SD], 5429.4 [5820.9] pg/mL; range, 27.0-23586.0 pg/mL) and positive results on RT-QuIC assays (66 of 71 patients [93%]). RT-QuIC assay results were negative or indeterminant in 5 patients. Initial brain MRI findings suggestive of CJD were reported in 88 of 115 patients (77%). The combined presence of characteristic MRI findings and CSF anomalies (either elevated T-tau level or positive results on RT-QuIC) identified all but 3 patients with probable or definite CJD at presentation (combined sensitivity, 107 of 110 patients [97%]). An additional 5 patients with an initial MRI inconsistent with CJD did not undergo CSF analyses (excluded from this analysis). Other findings were detected in substantially fewer patients, including myoclonus (31 of 115 patients [27%]), stereotyped EEG findings (ie, periodic discharges, 17 of 105 patients [16%]), and elevated CSF 14-3-3 protein level (54 of 90 patients [60%]).

**Table 2.** Clinical Features and Diagnostic Tests at Initial Presentation

Parameter	Patients, No. (%)			*P* value^a^
	All	Definite CJD (n = 40)	Probable CJD (n = 75)	
Clinical features				
Rapidly progressive dementia^b^	90 (77)	30 (75)	60 (80)	.64
Visual or cerebellar signs	74 (64)	25 (62.5)	49 (65)	.84
Myoclonus	31 (27)	10 (25)	21 (52.5)	.83
Pyramidal or extrapyramidal signs	29 (25)	14 (35)	15 (20)	.11
Akinetic mutism	4 (3)	2 (5)	2 (3)	.61
Periodic discharges or PSWC on EEG (n = 105)	17 (16)	9 (22.5)	8 (11)	.17
Brain MRI (n = 115)^c^				
Consistent with CJD	88 (77)	29 (72.5)	59 (79)	.49
Deep gray nuclei^d^	24 (21)	10 (25)	14 (19)	.37
Cortical ribboning	51 (44)	17 (42.5)	34 (45)	
Cortical and deep gray nuclei	13 (11)	2 (5)	11 (15)	
CSF analysis				
Protein 14-3-3	54/90 (60)	15 (52)	39 (64)	.27
T-tau >1149 pg/mL^e^	81/92 (88)	23 (79)	58 (92)	.09
RT-QuIC	66/71 (93)	21 (95)	45 (92)	.58

Abbreviations: CJD, Creutzfeldt-Jakob disease; PSWC, periodic sharp wave complexes; CSF, cerebrospinal fluid; T-tau, total tau; RT-QuIC, real time quaking-induced conversion test.

^a^ *P* values are based on χ^2^ test.

^b^ Rapidly progressive dementia at initial presentation considered positive if time from onset of progressive cognitive symptoms to assessment <12 months.

^c^ Brain MRI criteria as outlined within the Diagnostic Criteria by the Centers for Disease Control and Prevention (high signal in caudate or putamen or ≥2 cortical regions [temporal, parietal, occipital] either on diffusion-weighted imaging or fluid attenuated inversion recovery).^2^

^d^ Deep gray nuclei include hyperintensity in basal ganglia and/or thalamus involvement on T2- or diffusion-weighted imaging.

^e^ T-tau levels presented are from earliest T-tau sample available.

**Figure 1.**
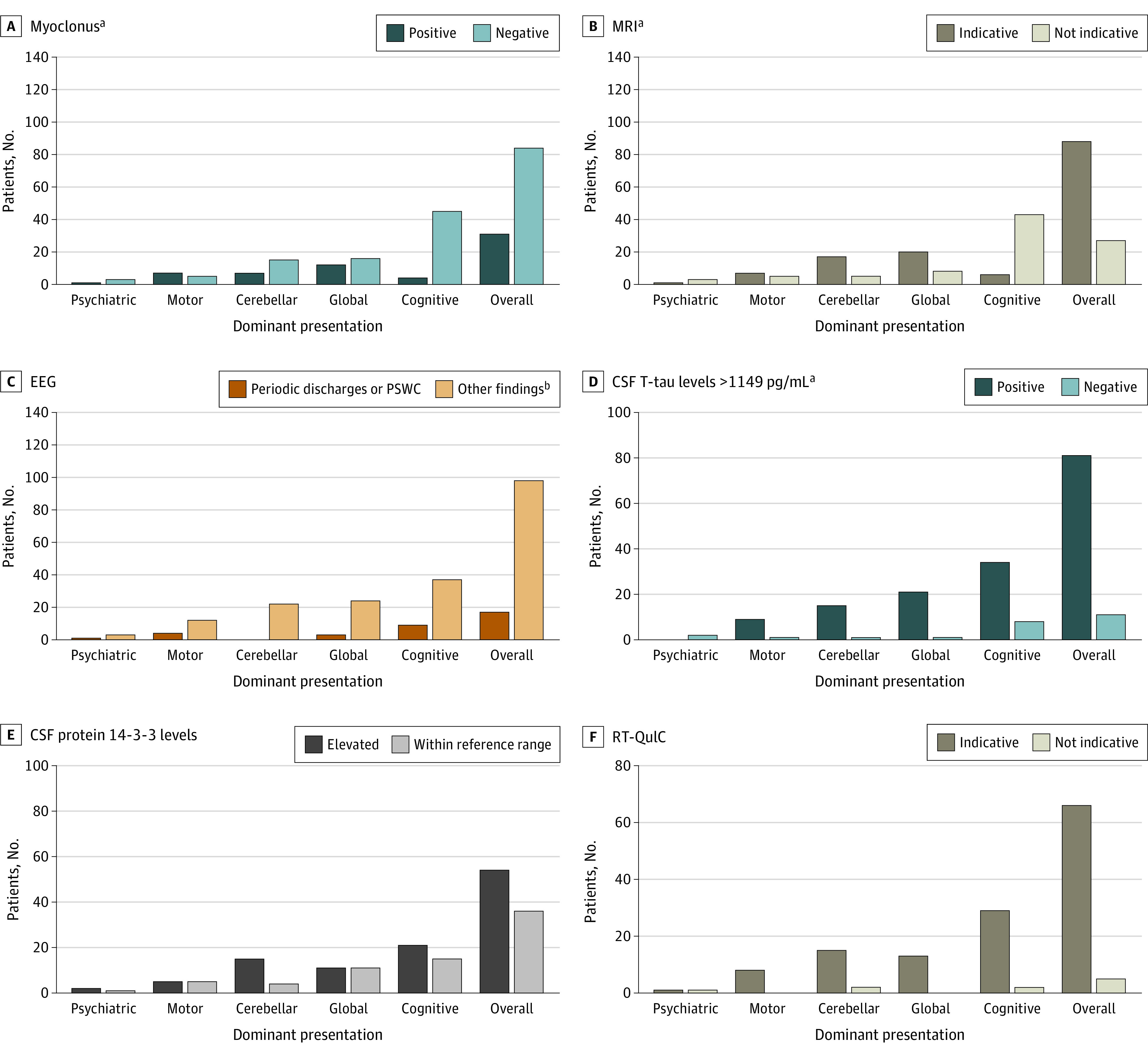
Diagnostic Features at Presentation According to Dominant Presentation in Creutzfeldt-Jakob Disease (CJD) ^a^*P* < .05. ^b^Other features on electroencephalography (EEG) include diffused slowing, epileptiform discharges, and normal tests. PSWC indicates periodic sharp wave complexes; RT-QuIC, real time quaking induced conversion test; T-tau, total tau.

Figure 1 depicts diagnostic features at presentation according to dominant presentation in CJD. MRI findings consistent with CJD were more common in patients with cognitive-predominant presentations (43 of 49 patients [88%]) vs motor-predominant presentations (7 of 12 patients [58%]; *P* = .03) and psychiatric-predominant presentations (1 of 4 patients [25%]; *P* = .01). Cortical ribboning was the most frequent anomaly in all subtypes except patients with cerebellar-predominant presentation, in whom deep gray nuclei (basal ganglia or thalamus) involvement predominated (eFigure 1 in the Supplement). Periodic discharges, including periodic sharp wave complexes, were limited to patients with cortically localized presentations and were not detected in any patients with cerebellar-predominant presentation (Figure 1). The sensitivity of CSF protein 14-3-3 detection was highest in the cerebellar group (15 of 19 patients [79%]) and lowest in the global (11 of 22 patients [50%]) and motor (5 of 10 patients [50%]) groups. CSF T-tau level greater than 1149 pg/mL was observed in 2 of 2 patients (100%) with psychiatric predominant presentations, 21 of 22 patients (95%) in the global group, 15 of 16 patients (94%) in the cerebellar group, and 34 of 42 patients (81%) in the cognitive group. Sensitivity of the RT-QuIC assay ranged from 15 of 17 patients (88%) among the cerebellar subgroup to 29 of 31 patients (94%) among the cognitive group. Only 2 patients in the psychiatric subgroup underwent CSF sampling; RT-QuIC results were positive in 1 patient.

### Diagnostic Yield of Repeat Testing

Findings consistent with CJD were not seen on initial brain MRI in 27 patients (23%). Neuroimaging was repeated a median (IQR) of 4.9 (2.9-12.1) weeks later in 24 patients; MRI findings were consistent with CJD in all patients with CJD. Of 20 patients without anomalous EEG findings at first assessment, 12 patients (60%) underwent repeat EEG, revealing anomalies in 9 patients (45%) (including periodic sharp wave complexes in 3 patients). The mean (SD) time between normal and anomalous EEG findings was 9.2 (7.4) weeks. Lumbar punctures were repeated in 3 of 11 patients (27%) with T-tau level less than 1149 pg/mL and 2 of 5 patients (40%) with negative or indeterminate RT-QuIC findings (2 patients had both negative or indeterminant RT-QuIC findings and subthreshold T-tau levels). T-tau levels were elevated to greater than 1149 pg/mL in 1 of 3 patients (33%) at repeat testing (interval between testing, 29 months), while RT-QuIC test results became positive in both patients (interval between testing, 8.7 and 4.5 weeks). An autopsy was completed in 1 patient whose RT-QuIC results were negative, confirming definite CJD. No repeated tests yielded a negative result following positive results.

### Clinical Findings, Biomarker Results, and Survival

**Figure 2.**
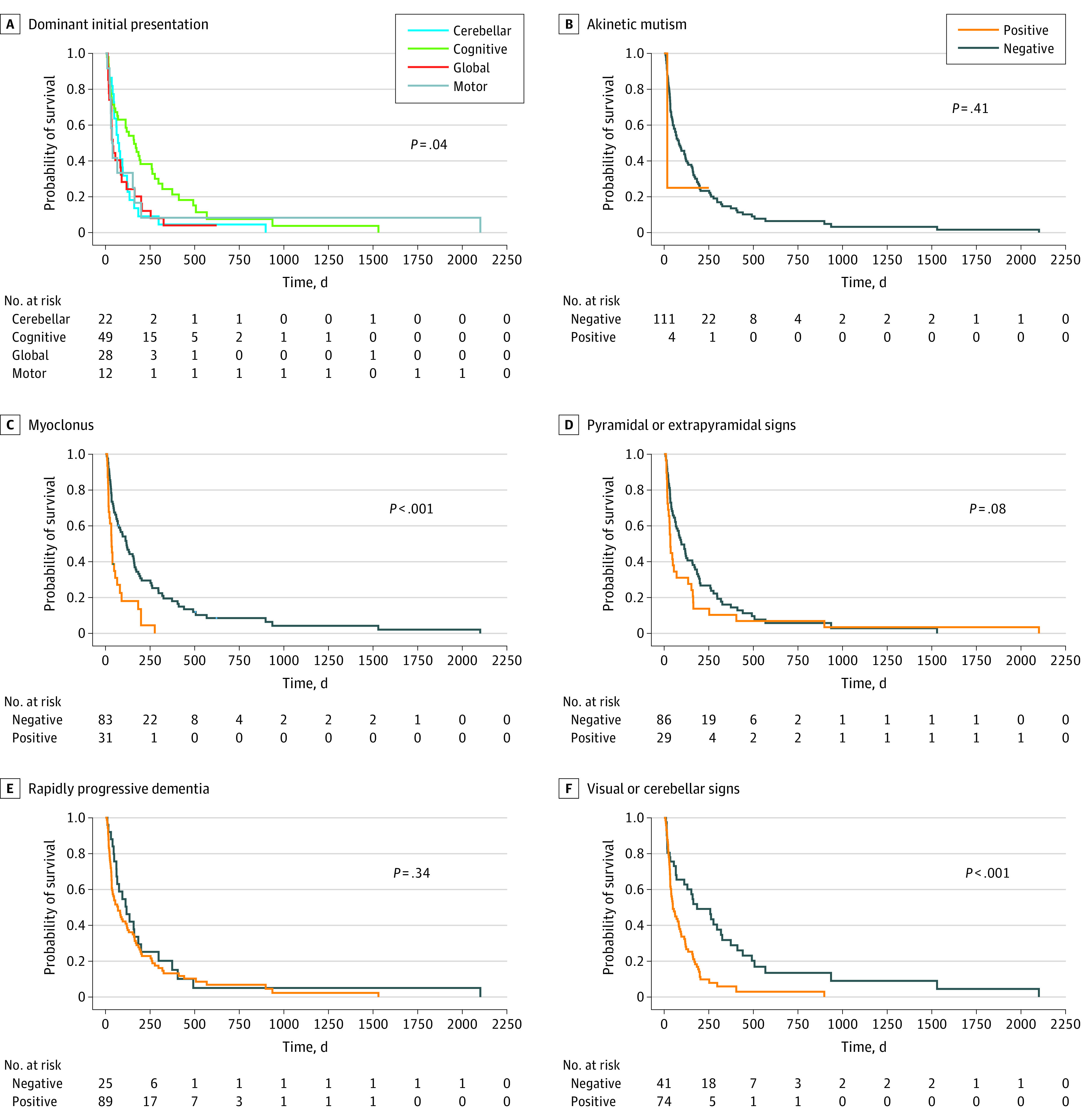
Survival Curves According to Clinical Features at Initial Presentation

**Figure 3.**
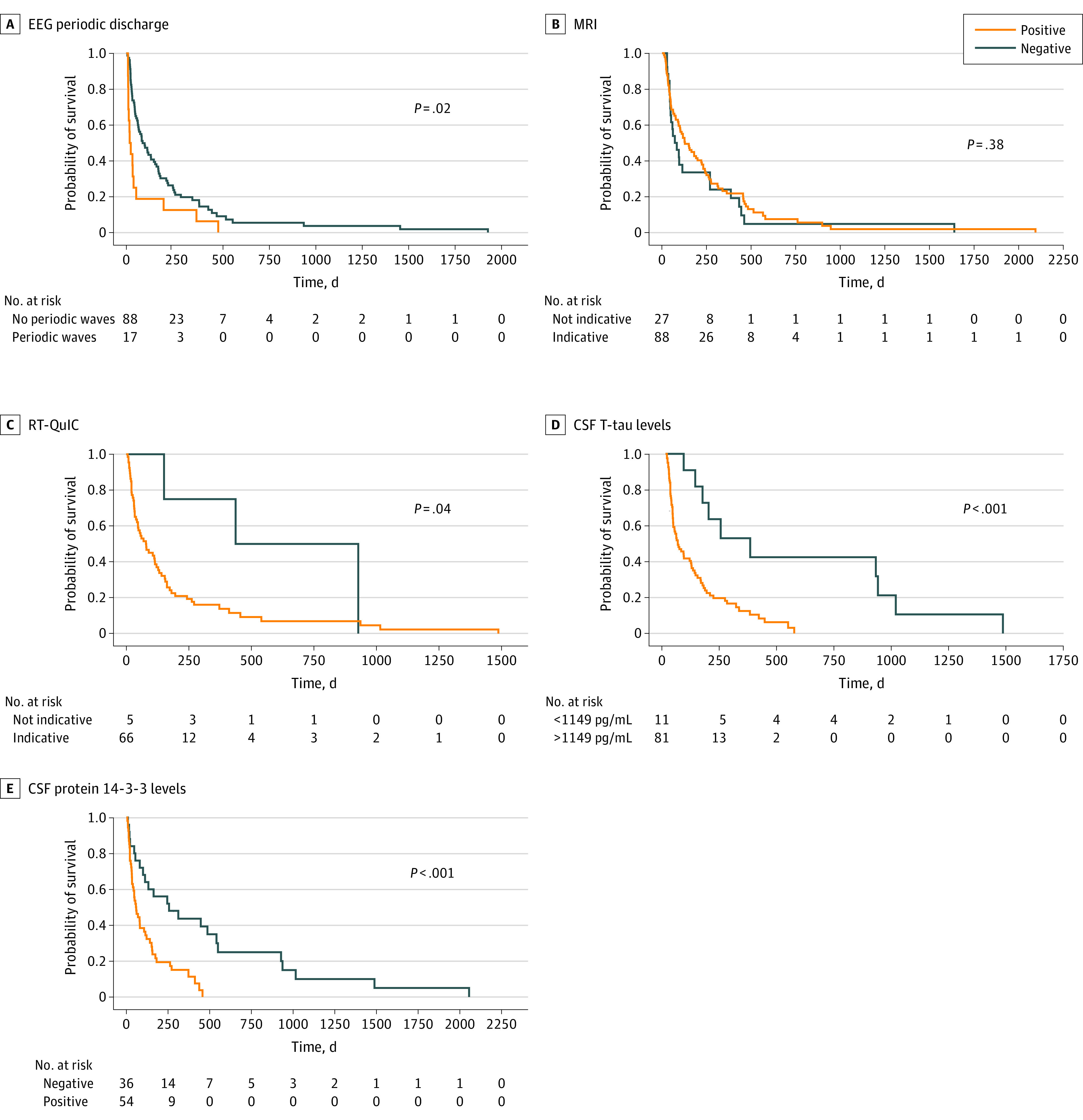
Survival Curves According to Diagnostic Tests at Initial Presentation CSF indicates cerebrospinal fluid; EEG, electroencephalography; MRI, magnetic resonance imaging; RT-QuIC, real time quaking induced conversion test; T-tau, total tau.

At the end of clinical follow-up (October 2021), 105 patients (91%) were deceased. Mean (SD) symptomatic duration was 359.3 (366.7) days (range, 27-2205 days). Figure 2 depicts Kaplan-Meier survival analyses stratified by predominant clinical presentations and clinical features at initial presentation. Mean survival was longest for patients with a cognitive predominant presentation (263.1 [95% CI, 154.7-371.5] days; *P* = .004). Patients with global impairment experienced the shortest mean survival (103.9 [95% CI, 51.6-156.1] days). The presence of myoclonus at presentation was associated with shorter mean survival compared with patients without myoclonus (67.8 [95% CI, 39.6-96.X] days vs 252.9 [95% CI, 158.6-347.2] days; χ^2^ = 15.83; *P* < .001). Visual or cerebellar signs at initial assessment, regardless of dominant presentation, were associated with shorter mean survival compared with patients without these signs (114.4 [95% CI, 72.9-155.9] days vs 377.6 [95% CI, 200.7-554.5] days; χ^2^ = 16.22; *P* < .001). The difference in survival between patients with pyramidal or extra-pyramidal symptoms at presentation vs patients without was not statistically significant (173.2 [95% CI, 23.7-322-7] days vs 208.9 [95% CI, 136.8-281.3] days; *P* = .08). Figure 3 depicts Kaplan-Meier survival analyses stratified by results of diagnostic tests at initial presentation. Detection of periodic discharges on the initial EEG was associated with shorter mean survival compared with patients without periodic discharges on initial EEG (104.5 [95% CI, 25.7-183.2] days vs 211.4 [95% CI, 152.7-309.8] days; χ^2^ = 5.913; *P* = .02). CSF T-tau levels greater than 1149 pg/mL were associated with a shorter mean survival compared with patients with lower T-tau levels (133 [95% CI, 97.3-168.6] days vs 579.6 [95% CI, 274.9-884.2] days; χ^2^ = 12.2; *P* < .001). Patients with elevated CSF protein 14-3-3 levels had shorter mean survival time compared with those without CSF protein 13-3-3 (124.5 [95% CI, 85.8-163.2] days vs 478.9 [95% CI, 253.8-704.0] days; χ^2^ = 13.3; *P* < .001). Negative or indeterminant RT-QuIC findings were associated with a longer mean survival (610.5 [95% CI, 211.7-1009.3] days vs 186.9 [95% CI, 135.5-298.9] days; χ^2^ = 4.2; *P* = .04). Mean survival did not differ based on detection of akinetic mutism, rapidly progressive dementia, or findings from the first MRI.

Multivariable linear regression was used to further evaluate the association of detection of clinical features and CSF biomarkers with the time to death (eTable in the Supplement). Patients with myoclonus had shorter survival time (difference, −125.9 [95% CI, −236.3 to −15.5] days; *P* = .03), as did those with visual or cerebellar signs (difference, −180.2 [95% CI, −282.2 to −78.2] days; *P* < .001). Additionally, elevated CSF protein 14-3-3 level was associated with a shorter disease duration (difference, −193 [95% CI, −304.9 to −82.9] days; *P* < .001), while each 1000 pg/mL increase in T-tau level was associated with decrease in time-to-death of 9.1 (95% CI, −17.7 to −1.0) days (*P* = .04). Although the timing of lumbar punctures varied widely (mean [SD] time from symptom onset to lumbar puncture, 179 [190] days; range, 1-1052 days), T-tau levels were not associated with days from symptom onset (Pearson ρ = −0.144; *P* = .17), suggesting that T-tau levels reflected disease activity or severity, not duration.

## Discussion

This cohort study considered the diagnostic performance of key clinical features and widely available tests in patients with CJD diagnosed following the advent of RT-QuIC testing. Although myoclonus and periodic discharges on EEG are historically associated with CJD,^21^ the diagnostic yield of these features was low in this cohort. By contrast, the diagnostic performance of CSF tests for CJD (RT-QuIC assays and T-tau level greater than 1149 pg/mL) and stereotyped signal anomalies on MRI was high, consistent with prior publications.^15,22^ Indeed, the combination of RT-QuIC, T-tau level greater than 1149 pg/mL, and stereotyped MRI findings identified all but 3 patients (97%) with CJD in this series. No differences in sensitivity of clinical features or diagnostic test results were observed between patients with definite vs probable diagnoses, further validating the diagnostic criteria for probable CJD. The detection of myoclonus or visual or cerebellar findings was associated with shorter disease duration, as were elevations of CSF protein 14-3-3 and T-tau levels, suggesting that these variables may inform disease prognosis. Collectively, these findings may guide prioritization and interpretation of accessible tests in patients with suspected CJD evaluated in the modern era.

Implementation of RT-QuIC testing may allow earlier and more accurate diagnoses of patients with a broader spectrum of clinical symptoms and signs attributable to CJD,^14,15^ but this test is not perfect. Owing to the complexities involved in running this assay, including prolonged agitation of sample and medium, diagnostic and reporting delays are common. The test may also yield false negative or indeterminant results, as observed in small numbers of patients in this study and others.^13,15,23^ For these reasons, additional diagnostic biomarkers should be measured in patients with suspected CJD and selected tests (ie, MRI, CSF T-tau, and RT-QuIC) should be repeated when diagnostic suspicion remains high in the face of initial negative or indeterminant test results. MRI is commonly used in the diagnostic evaluation of suspected CJD, and for good reason.^24^ The MRI sensitivity rate reported in this study at presentation is consistent with that reported in similar studies (39.1%-92.3%).^4,9,25^ These results affirm the value of MRI in the evaluation of patients with early symptomatic CJD. Yet it is important to highlight that among the psychiatric dominant initial presentation, MRI sensitivity was low, further adding to the diagnostic challenge and risk of diagnostic errors in this clinical subtype. Importantly, repeated scans later in the disease course had high yield, with 112 of 115 patients in this cohort eventually having at least 1 brain MRI result consistent with CJD.

Although elevated CSF protein 14-3-3 level has historically been viewed as a useful marker of CJD, our results suggest that this biomarker may have limited diagnostic application in the modern era. Previous studies report a wide range in diagnostic sensitivity of CSF protein 14-3-3 level, ranging from low (53%^26^) to high (85%-92%^9,11,27^) across cohorts. The poor sensitivity (60% in this study) and specificity (elevated CSF protein 14-3-3 levels have been reported in patients with viral encephalitis, hypoxic encephalopathy, metastases, and metabolic and paraneoplastic syndromes^27,28,29,30^) suggest that elevated CSF protein 14-3-3 levels have been supplanted by CSF measures of T-tau (affirmed in this study and others^17^) and RT-QuIC. Diagnostic algorithms should be adjusted accordingly.

Beyond informing diagnoses, biomarkers may have prognostic implications. Indeed, elevations in CSF T-tau and protein 14-3-3 levels were independently associated with time from testing to death in this series. T-tau and CSF protein 14-3-3 levels are presumed to reflect rapid neuronal damage,^31,32^ providing a logical mechanism underpinning this relationship in CJD, and justifying continued measurement of CSF protein 14-3-3 levels in clinical cohorts with suspected CJD.

### Limitations

This study has some limitations. The study was performed at tertiary care centers, using retrospective data. Under this rubric, selected CJD cases could have been missed or misdiagnosed at our center, or selected tests results (eg, MRI findings) could have been misinterpreted. The use of RT-QuIC results as a diagnostic criterion for probable CJD may have led to unintentional exclusion of patients and overestimation of the sensitivity of RT-QuIC for the diagnosis of CJD. Collectively, these limitations may have affected our estimates of the diagnostic sensitivity of clinical features and tests. We must also acknowledge that we intentionally focused on variables that informed diagnostic criteria to the exclusion of others. Thus, our results do not inform optimal testing for diagnosis and prognosis in CJD, but rather the use of accessible clinical features and biomarkers. These factors limit generalizability of our study results and necessitate replication in other populations. While we focused on initial presentation, it is also important to acknowledge that detection of these biomarkers might increase throughout the disease course, as seen in individuals who underwent repeat MRIs. Prospective studies, including standardized clinical assessments with longitudinal measurement of established and emergent clinical features and biomarkers (eg, neurofilament light chain,^33,34^ phosphorylated tau^35,36^), are needed to further inform the care of patients with CJD.

## Conclusions

The findings of this cohort study suggest that CSF RT-QuIC, elevated T-tau level, and stereotyped MRI anomalies continue to support the diagnosis of CJD in the modern era, while other clinical findings (eg, myoclonus) and biomarkers traditionally ascribed to CJD (eg, periodic sharp wave complexes on EEG, CSF protein 14-3-3 level) offer less diagnostic value owing to high false negative potential. Detection of visual or cerebellar features, myoclonus, and CSF protein 14-3-3 and T-tau levels may be associated with disease duration, justifying inclusion in the evaluation of patients with suspected CJD. Comprehensive evaluation incorporating multiple diagnostic biomarkers is important to optimize diagnosis and inform survival in patients with CJD.

## Notes

Supplement. **eTable.** Multivariable Regression Assessing Association of Clinical Features and CSF Biomarkers With Disease Duration **eFigure.** MRI and EEG Findings in CJD According to Dominant Presentation
