# Supplementary material for: Analysis of Clinical Features, Diagnostic Tests, and Biomarkers in Patients With Suspected Creutzfeldt-Jakob Disease, 2014-2021
Source: JAMA Netw Open. 2022 Aug 3;5(8):e2225098. doi: 10.1001/jamanetworkopen.2022.25098 (PMC9350714; doi:10.1001/jamanetworkopen.2022.25098)
Supplement: Supplement. — eTable. Multivariable Regression Assessing Association of Clinical Features and CSF Biomarkers With Disease Duration eFigure. MRI and EEG Findings in CJD According to Dominant Presentation [file jamanetwopen-e2225098-s001.pdf]

Supplemental Online Content

Shir D, Lazar EB, Graff-Radford J, et al. Analysis of clinical features, diagnostic tests, and biomarkers in patients with suspected Creutzfeldt-Jakob disease, 2014-2021. *JAMA Netw Open*. 2022;5(8):e2225098. doi:10.1001/jamanetworkopen.2022.25098

eTable. Multivariable Regression Assessing Association of Clinical Features and CSF Biomarkers With Disease Duration

eFigure. MRI and EEG Findings in CJD According to Dominant Presentation

This supplemental material has been provided by the authors to give readers additional information about their work.

© 2022 Shir D et al. *JAMA Network Open.*

eTable. Multivariable Regression Assessing Association of Clinical Features and CSF Biomarkers With Disease Duration

**Clinical Feature/Diagnostic Test**				
	n [a]	β	p. value	95% CI
**Model 1**				
Myoclonus	115	-125.87	**0.026**	-236.3, -15.49
Visual/Cerebellar signs		-180.19	**<0.001**	-282.2, -78.18
**Model 2**				
14-3-3 positive	147	-193.92	**<0.001**	-304.9, -82.9
T-tau, pg/mL		-0.009	**0.041**	-0.018, -0.001
RT-QuIC positive		-49.59	0.592	-232.6, 133.4

Linear regression, Model 1 controlling for age and visual/cerebellar signs, myoclonus. Model 2 controlling for age and "positive" protein 14-3-3, t-tau levels and RT-QuIC. [a] N for Myoclonus and Visual/Cerebellar signs was based on the total number of patients included in the analyses; n for CSF biomarkers includes total of CSF samples included in the analyses.
Abbreviations: T-tau, total tau. RT-QuIC, real time quaking induced conversion test.

© 2022 Shir D et al. *JAMA Network Open.*

eFigure. MRI and EEG Findings in CJD According to Dominant Presentation

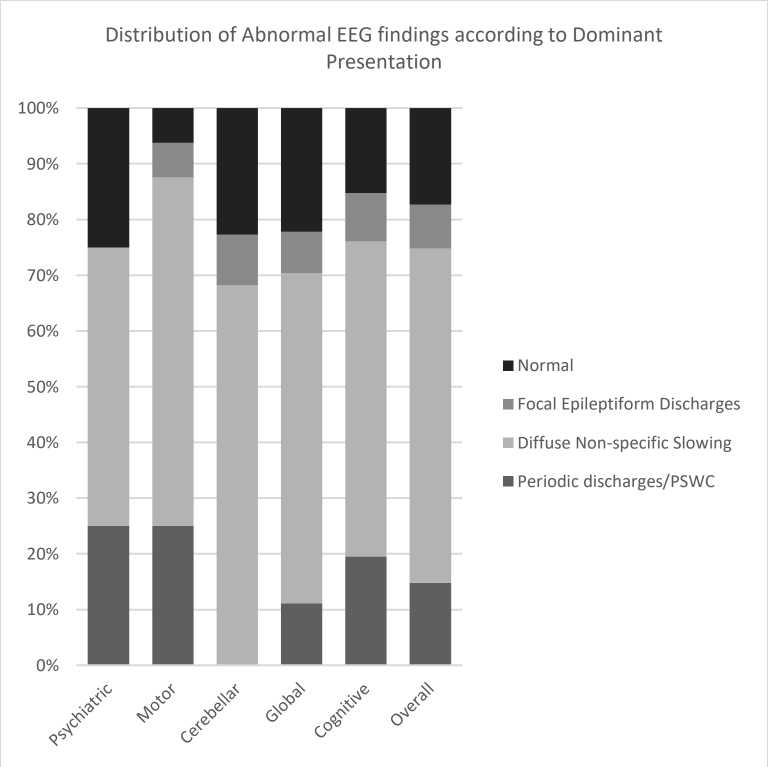

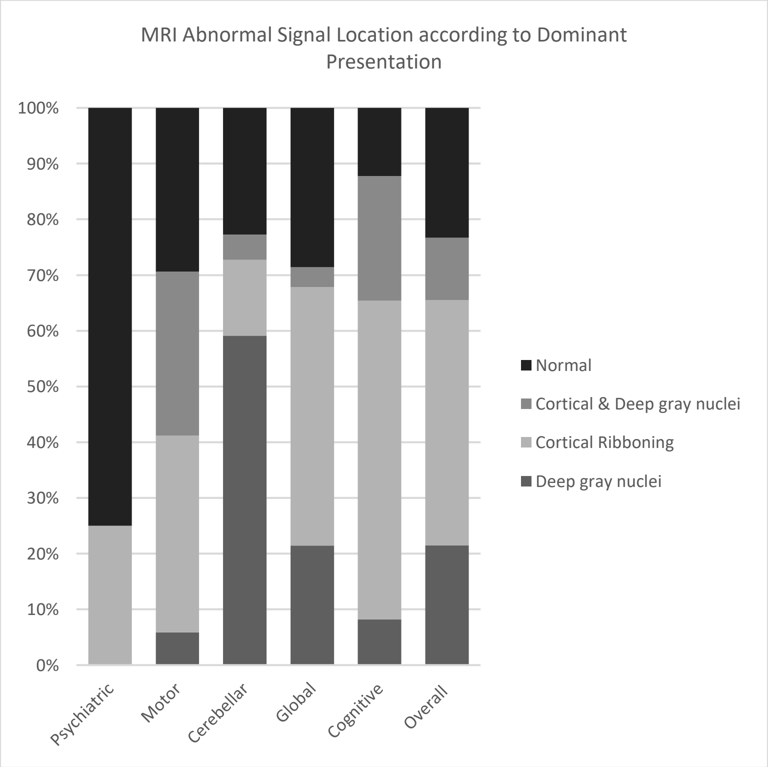

© 2022 Shir D et al. *JAMA Network Open.*
